# Biodiversity in targeted metabolomics analysis of filamentous fungal pathogens by ^1^H NMR-based studies

**DOI:** 10.1007/s11274-017-2285-7

**Published:** 2017-06-05

**Authors:** Adam Ząbek, Magdalena Klimek-Ochab, Ewa Jawień, Piotr Młynarz

**Affiliations:** Department of Chemistry, Wroclaw University of Technology, Wybrzeże Wyspiańskiego 27, 50-370 Wrocław, Poland

**Keywords:** Metabolomics, ^1^H NMR spectroscopy, Biodiversity, Filamentous fungal pathogen

## Abstract

**Abstract:**

The taxonomical classification among fungi kingdom in the last decades was evolved. In this work the targeted metabolomics study based on ^1^H NMR spectroscopy combined with chemometrics tools was reported to be useful for differentiation of three model of fungal strains, which represent various genus of Ascomycota (*Aspergillus pallidofulvus, Fusarium oxysporum, Geotrichum candidum*) were selected in order to perform metabolomics studies. Each tested species, revealed specific metabolic profile of primary endo-metabolites. The species of *A. pallidofulvus* is represented by the highest concentration of glycerol, glucitol and Unk5. While, *F. oxysporum* species is characterised by increased level of propylene glycol, ethanol, 4-aminobutyrate, succinate, xylose, Unk1 and Unk4. In *G. candidum*, 3-methyl-2-oxovalerate, glutamate, pyruvate, glutamine and citrate were elevated. Additionally, a detailed analysis of metabolic changes among *A. pallidofulvus, F. oxysporum* and *G. candidum* showed that *A. pallidofulvus* seems to be the most pathogenic fungi. The obtained results demonstrated that targeted metabolomics analysis could be utilized in the future as a supporting taxonomical tool for currently methods.

**Graphical Abstract:**

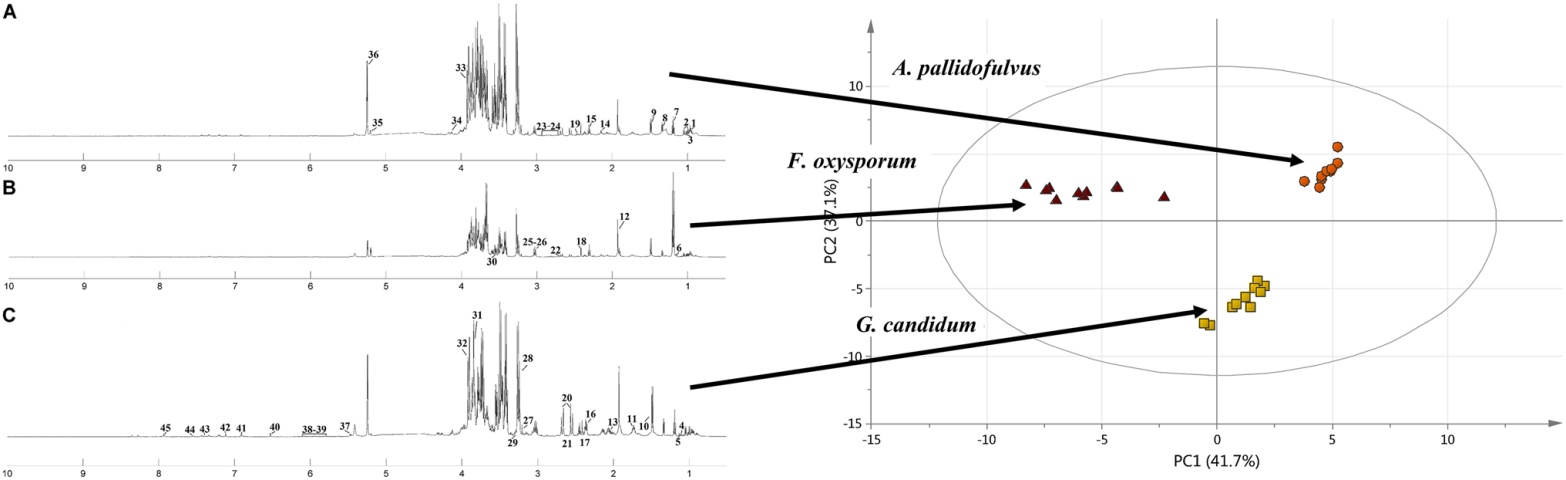

## Introduction

The old taxonomic approach for fungi was generally based on phenotype (Guarro et al. 1999). This approach included comparative studies of the morphological structures, cell wall composition, cytological testing, ultrastructure, cellular metabolism, fossil record, and sexual cycle (Bartnicki-Garcia 1970, 1987; Fuller 1976; Hawksworth et al. 1995; Heath 1980, 1986; Le´John 1974; Taylor 1978; Vogel 1964). According to this method, the fungi kingdom was divided into the following four main phyla: *Zygomycota, Ascomycota, Basidomycota* and *Fungi Imperfecti*, namely *Deuteromycota* (Guarro et al. 1999). This division has changed during the last two decades, especially due to the development of new cladistics and molecular approaches (phylogenetic), such as a PCR method in which universal oligonucleotide primers specific to fungi were selected and the 18S subunits of the rRNA sequences were compared (Bruns et al. 1992; Golenberg et al. 1990; Hendriks 1992; Hausner et al. 1992; Haase et al. 1995; Spatafora and Blackwell 1993; Swann and Taylor 1995). Currently, the classification system of fungi contains the following seven phyla: *Chytridiomycota, Blastocladiomycota, Neocallimastigomycota, Microsporidia, Glomeromycota, Ascomycota* and *Basidiomycota* (Kirk et al. 2001, 2008; Hibbett et al. 2007; Blackwell et al. 2006; David et al. 2011). It can be imagined that this systematic scheme might further evolve in the future because of the huge biodiversity of fungal species and techniques that are still being developed.

The procedure of microbial identification can also be based on the analysis of the chemical compositions of cells (Ivanisˇevic´ et al. 2011; Semmar et al. 2007; Cevallos-Cevallosa et al. 2009). This approach is successfully used to screen for metabolic differences of various living systems. For example, Zieliński et al. reported that chemometrics studies are useful for determination of the origin of polish monofloral and multifloral honeys, whereas Deja et al. showed that multivariate analysis of primary endo-metabolites can exhibit correlation between fruit bodies and the topsoil type as well as differences in the chemical compositions of the stem and cap of *Amanita muscaria* (Zieliński et al. 2014; Deja et al. 2014). Apart from a few papers showing the possibility of organism classification according to their intracellular metabolite compositions, most of literature data demonstrated differentiation power based on secondary endo-metabolites (Frisvad et al. 2008; Andersen et al. 2008; Frisvad 1992; Jennessen et al. 2005). Generally, this trend could be observed in studies on fungal biodiversity, where chemotaxonomy is referred to as being successful for its classification. Metabolomics approaches have proven to be useful to distinguish among genera such as *Penicilium, Aspergillus* and *Fusarium* (Larsen et al. 2005; Smedsgaard and Nielsen 2005). Kadlec et al. exhibited differentiation between *Tolypocladium, Beauceria* and *Paecilomyces* by using gas chromatography combined with mass spectrometric analysis (Kadlec et al. 1994). Moreover, chemotaxonomic diversity among *Saccharomyces cerevisiae* mutant groups was observed (Smedsgaard and Nielsen 2004; Smedsgaard et al. 2004; Mas et al. 2007). Although chemotaxonomic studies associated with a metabolomics approach have been conducted based on secondary endo-metabolites, they have rarely been conducted on primary endo-metabolites. A few reports could be found in which components of the grow medium (metabolic footprint) were used for classification purposes (Junka et al. 2013; Zheng et al. 2011).

The main goal of this study was to examine a potential metabolomics-based approach for the supporting currently taxonomy of filamentous fungal pathogens by applying ^1^H NMR spectroscopy in association with chemometric analysis. The common fungal human pathogens *Aspergillus pallidofulvus* (*A. pal*), *Fusarium oxysporum* (*F. oxy*) and *Geotrichum candidum* (*G. can*) were chosen as model microorganisms. These fungal species belong to three different classes (*Eurotiomycetes, Sordariomycetes*, and *Saccharomycetes*) of the *Ascomycota* phylum. It should be noted that *Eurotiomycetes* and *Sordariomycetes* come from the same subphylum *Pezizomycotina*, whereas *Saccharomycetes* come from *Saccharomycotina*. With regard to previous reports, this is the first study in which the simple distinguish of filamentous fungi based on primary not secondary metabolites was observed. Moreover, the second objective was to determine characteristic metabolites related to the occurrence of the tested fungal pathogens. Finally, the specific biochemical pathways were discussed.

## Materials and methods

### Fungal strains and culture conditions

In this study, three genus of filamentous fungi including *Aspergillus pallidofulvus* (ZK0431), *Fusarium oxysporum* (DSM 12646) and *Geotrichum candidum* (DSM 6593) were tested. Fungal strains were routinely maintained on potato dextrose agar (PDA, Difco), which provided profuse sporulation suitable for collection of the inoculum.

To evaluate the differences and biodiversity between *Aspergillus pallidofulvus, Fusarium oxysporum* and *Geotrichum candidum* based on intracellular metabolites, fungi were cultured on potato dextrose agar (PDA, Difco) in 10-cm Petri dishes for 5 days at 28 °C. The inoculums of fungi were prepared by washing with 5 mL of 0.1% Tween 20. The spore suspensions were adjusted to a final concentration of 10^6^ conidia/mL in 100 mL of potato dextrose broth (PDB, Difco) and incubated with shaking for 48 h at 28 °C.

### Extraction of the fungal metabolites

The fungal biomasses were filtered, washed with saline solution and then weighed in aliquots of 100 mg of wet weight cells. In the next step, fungal cells were frozen for 10 min at −80 °C. The procedure of fungal cell disintegration included two separate consecutive steps. In the first one, frozen samples were disrupted using QIAGEN–TissueLyser (50 Hz, 5 min), followed by the addition of 650 µL of PBS buffer (10% D_2_O, 0.05 M, pH = 7.0, 0.15 mM TSP) to each sample. The second step based on ice bath ultrasonic cell disruption was performed for 30 min. After the disintegration procedure was completed, samples were centrifuged (10 min, 17 500 rpm, 4 °C), and 550 µL of clarified homogenate was transferred into a 5 mm NMR tube. Ten repetitions for each strain were performed in this study.

### ^1^H NMR spectroscopy analysis of the fungal metabolites

Standard NMR experiments were performed on a Bruker AVANCE II 600.58 MHz spectrometer equipped with a 5 mm TBO probe at 300 K. All one-dimensional ^1^H NMR spectra were carried out using the *zgpr1d* (in Bruker notation) pulse sequence by suppression of water resonance by presaturation. Acquisition parameters were as follows: spectral width, 20 ppm; acquisition time, 1.36 s per scan; time domain points, 32 K; relaxation delay, 3.5 s; and number of scans, 256. Prior to Fourier transformation, the FIDs were multiplied by an exponential function equivalent to that of a 0.3 Hz line-broadening factor. The spectra were manually corrected for phase and baseline (by a fifth order polynomial baseline fitting) and referenced to the TSP resonance at 0.0 ppm. Additionally, the metabolite conformations were assigned through two-dimensional NMR experiments ^1^H-^1^H-TOCSY (Total Correlation Spectroscopy) and ^1^H-^13^C-HSQC (Heteronuclear Single Quantum Coherence).

### Data processing and multivariate statistical data analysis

All spectra were exported to Matlab (Matlab v. 8.1, Mathwork Inc.) for preprocessing. Regions affected by solvent suppression were excluded (4.500–5.100 ppm) and alignment procedures involving the correlation of optimized warping (COW) and interval correlation shifting (*i*coshift) algorithms were applied (Tomasi et al. 2004; Savorani et al. 2010). The spectra consisted of 30,811 data points and were normalized using the probabilistic quotient method to overcome the issue of dilution (Dieterle et al. 2006).

The multivariate and statistical data analysis were performed on a set of the 51 assigned metabolites. The metabolite concentration measured by NMR was obtained as the sum of the intensities of the non-overlapping resonances (or a cluster of partly overlapping resonances). Such a transformed data matrix was the input for SIMCA-P software (v 13.0, Umetrics, Umeå, Sweden) and Matlab for follow-up analysis.

Prior to the chemometric analysis, the data sets were unit variance scaled. For classification of the fungal strains, principal component analysis (PCA) was carried out. The fungal strains were assigned to groups according to the results of the homologous metabolite profiling that coincided with those of PCA-hierarchical cluster analysis (PCA-HCA). The graphical representation of the metabolite and fungal strains biplot was constructed from the first two components. The scores and loadings in the biplot were expressed using correlation scaling. A heat map with dendrograms to show dynamic changes in metabolites in the hierarchical clustering of data was created.

Metabolites responsible for the separation in models were tested using STATISTICA 10 with the Mann–Whitney–Wilcoxon test (MWW). A 0.01 level of probability was used as the criterion for statistical significance. Correlation coefficients (r) were also calculated.

## Results

**Fig. 1:**
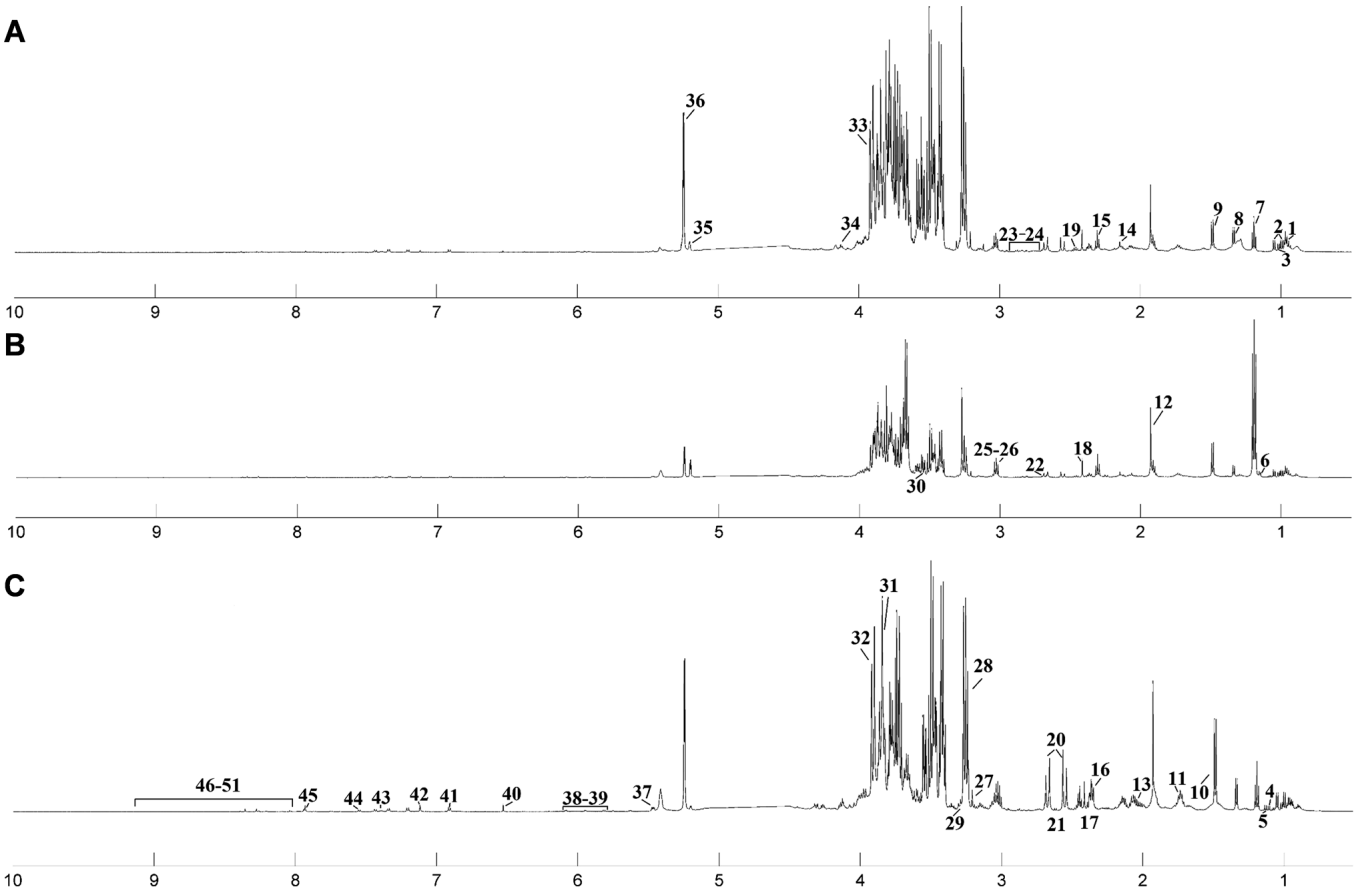
Median 600 MHz ^1^H NMR zgpr spectra of filamentous fungal pathogen strains obtained from cell-free crude extracts of the following: **a** *Aspergillus pallidofulvus*; **b** *Fusarium oxysporum*; **c** *Geotrichum candidum. 1* leucine; *2* valine; *3* isoleucine; *4* isobutyrate; *5* 3-methyl-2-oxovalerate; *6* propylene glycol; *7* ethanol; *8* lactate; *9* alanine; *10* arginine; *11* lysine; *12* acetate; *13* proline; *14* methionine; *15* 4-aminobutyrate; *16* glutamate; *17* pyruvate; *18* succinate; *19* glutamine; *20* citrate; *21* 2-oxoisocaproate; *22* malate; *23* aspartate; *24* asparagine; *25* creatine; *26* malonate; *27* CHOLINE; *28* sn-glycero-3-phosphocholine; *29 myo*-inositol; *30* glycerol; *31* glucitol; *32* mannitol; *33* betaine; *34* threonine; *35* xylose; *36* glucose; *37* glucose-1-phosphate; *38* uracil; *39* guanosine; *40* fumarate; *41* tyrosine; *42* histidine; *43* phenylalanine; *44* tryptophan; *45* xanthine; *46* Unk1; *47* Unk2; *48* Unk3; *49* Unk4; *50* Unk5; *51* Unk6

Representative ^1^H NMR-spectra for fungal strains are shown in Fig. 1. In total, 45 metabolites were identified based on typical 2D NMR experiments, namely ^1^H-^1^H-TOCSY and ^1^H-^13^C-HSQC. All assignments were verified using the Biological Magnetic Resonance Data Bank (BMRDB) and the Human Metabolome Data Base (HMDB) (Andersen et al. 2001, 2003). Six metabolites were not assigned (Unk1, Unk2, Unk3, Unk4, Unk5, Unk6).

### Multivariate analysis of the metabolite fingerprinting in *A. pallidofulvus, F. oxysporum* and *G. candidum*

**Fig. 2:**
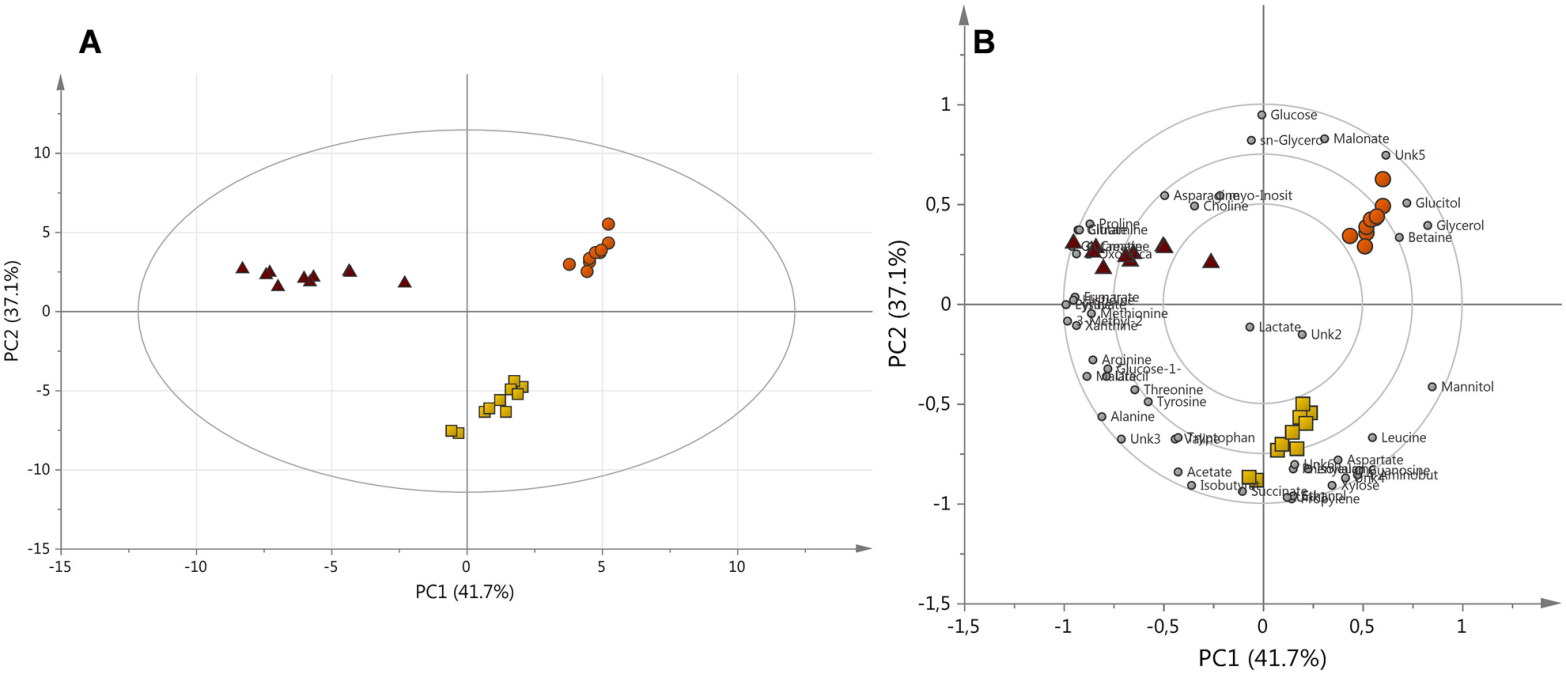
**a** PCA PC1/PC2 score plots of ^1^H NMR of the 51 total assigned metabolites and **b** Clusters in a two-dimensional biplot for *A. pallidofulvus* (*orange circles*), *F. oxysporum* (*yellow* boxes) and *G. candidum* (*red* triangles). (Color figure online)

In general, PCA was calculated with four principal components (PC) and revealed the natural grouping of the various fungal strains based on concentrations of the assigned endo-metabolites (Fig. 2a).

**Fig. 3:**
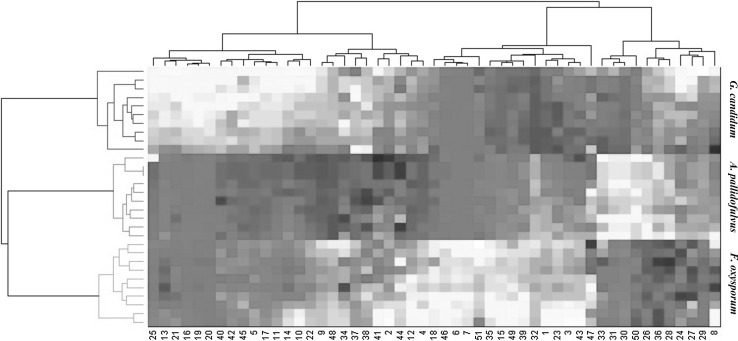
Dendrogram of HCA results obtained from PCA based on 4 PCs

In agreement with PCA, PCA-HCA applied on the same dataset revealed consistent resolution of the fungal strains (Fig. 3). The hierarchical clustering of the metabolite data showed three major groups of samples that were similar (*A. pallidofulvus, F. oxysporum* and *G. candidum*) to those of PCA. As shown in Fig. 2a, G. *can* is clearly separated from the other fungal genus. Among the other genus, *A. pal* and *F. oxy* were found to be the most similar to themselves.

The first and second PC (PC1 and PC2, respectively) accounted for 41.7% and 37.1% of the variance in the data, respectively. The data points of *A. pal* and *F. oxy* were separated from *G. can* in PC1, whereas differences between *A. pal* and *F. oxy* were in PC2 (Fig. 2a).

**Fig. 4:**
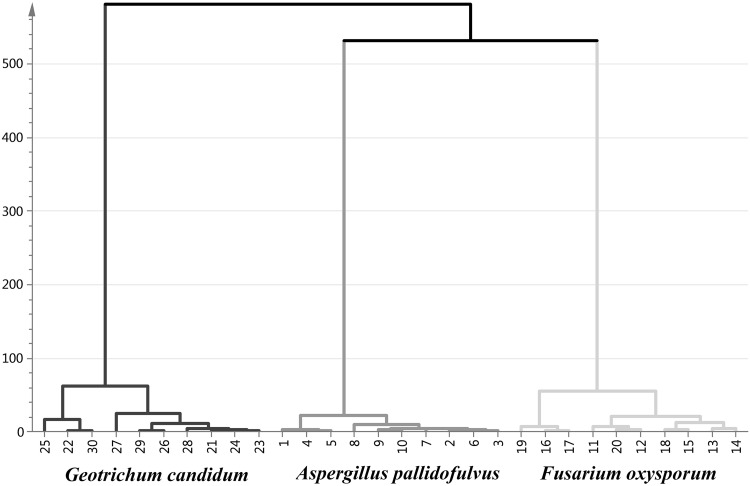
The heat map with hierarchical clustering of the metabolites. The metabolites assigned in columns are labeled with the same numbering scheme as in Fig. 1

The dynamic changes in the metabolite concentrations in the three clusters were explored using a heat map with hierarchical clustering (Fig. 4). The cluster of *A. pal* was characterized by increased levels of malonate, choline, sn-glycero-3-phosphocholine, glycerol, glucitol, betaine, glucose and Unk5. The group of *F. oxy* showed higher levels of leucine, isoleucine, isobutyrate, propylene glycol, ethanol, acetate, 4-aminobutyrate, succinate, aspartate, xylose, guanosine, phenylalanine, Unk1, Unk4 and Unk6.

In *G. can* the concentrations of 3-methyl-2-oxovalerate, alanine, proline, glutamate, pyruvate, glutamine, citrate, 2-oxoisocaproate, creatine, fumarate, histidine and xanthine were significantly higher.

The statistical analysis performed by using the MWW exhibited that in every possible comparison (*A. pal* vs. *F. oxy, A. pal* vs. *G. can, F. oxy* vs. *G. can*), almost every metabolite was statistically significant (Table 1).

**Table 1:** The changes in intracellular primary endo-metabolites

Metabolite	Percentage difference			Relative standard deviation (%)		
	*A. pal* vs. *F. oxy*	*A. pal* vs. *G. can*	*F. oxy* vs. *G. can*	*A. pal*	*F. oxy*	*G. can*
Leucine	−20.6*	44.3*	63.5*	13.0	11.2	15.0
Valine	−28.0*	−18.0	10.2	14.5	10.1	15.8
Isoleucine	−29.2*	13.0	41.8*	13.1	10.1	14.4
Isobutyrate	−75.8*	−47.4*	31.2*	7.3	8.1	10.2
3-Methyl-2-oxovalerate	−46.5*	−83.0*	−40.3*	3.6	11.8	11.5
Propylene glycol	−147.3*	−5.6	144.6*	5.0	12.4	8.2
Ethanol	−171.8*	−4.4	170.6*	24.6	30.2	14.9
Lactate	1.0	3.9	2.8	5.8	11.7	14.0
Alanine	−74.2*	−77.1*	−3.4	8.9	11.7	9.3
Arginine	−17.8*	−26.7*	−9.0	5.0	10.8	10.2
Lysine	−26.7*	−62.2*	−37.0*	9.9	10.8	11.0
Acetate	−75.0*	−51.4*	26.2*	9.7	10.0	15.8
Proline	7.1	−32.2*	−39.1*	4.9	10.3	11.1
Methionine	−9.8	−26.4*	−16.7	8.5	11.6	12.5
4-Aminobutyrate	−66.1*	82.1*	130.5*	6.1	10.0	9.2
Glutamate	−9.8	−92.7*	−84.8*	9.0	13.1	9.9
Pyruvate	−31.0*	−66.8*	−37.7*	5.4	10.7	6.9
Succinate	−61.5*	−19.5*	43.2*	5.4	11.8	8.8
Glutamine	5.7	−81.7*	−86.4*	3.7	11.5	8.3
Citrate	4.4	−97.3*	−100.6*	4.2	12.2	7.1
2-Oxoisocaproate	−5.6	−46.1*	−40.8*	5.5	13.5	8.0
Malate	−36.4*	−46.7*	−10.7	5.2	14.7	7.9
Aspartate	−27.2*	23.0*	49.4*	8.9	16.9	10.8
Asparagine	12.5*	−7.5	−19.9*	6.1	12.2	8.9
Creatine	−6.2^*^	−67.0*	−61.4*	40.7	11.1	9.6
Malonate	57.9*	28.9*	−30.2*	5.1	13.0	8.4
Choline	18.8	−3.9*	−22.6	5.0	18.6	17.9
sn-Glycero-3-phosphocholine	32.2*	6.3	−26.0*	6.8	10.1	9.0
*myo*-Inositol	33.2*	−0.1	−33.2*	5.1	35.9	7.6
Glycerol	74.7*	116.3*	53.1*	6.9	34.1	9.0
Glucitol	55.3*	65.0*	10.7	6.1	22.9	7.0
Mannitol	−5.4	106.3*	110.1*	6.9	9.6	8.8
Betaine	18.6*	26.5*	8.0	7.2	7.4	9.4
Threonine	−19.5*	−21.6*	−2.2	8.7	8.2	11.6
Xylose	−80.6*	44.8*	115.0*	10.2	24.6	6.4
Glucose	68.3*	9.9	−59.4*	8.2	6.6	15.8
Glucose-1-phosphate	−61.3*	−79.0*	−20.1	13.0	21.0	11.7
Uracil	−50.3*	−63.5*	−14.3	21.0	6.9	15.9
Guanosine	−44.0*	45.3*	85.0*	12.9	10.0	10.4
Fumarate	−33.3*	−73.6*	−43.0*	15.6	9.6	12.9
Tyrosine	−15.7	−15.4*	0.3	12.6	6.5	9.5
Histidine	−32.9*	−73.7*	−43.4*	12.0	17.6	9.1
Phenylalanine	−22.4*	5.5	27.9*	10.9	8.1	11.0
Tryptophan	−30.7*	−22.8*	8.0	16.0	8.1	7.8
Xanthine	−85.3*	−123.8*	−52.3*	13.6	23.7	20.4
Unk1	−121.7*	−5.8	118.0*	17.6	17.1	11.6
Unk2	−0.8	6.2	7.0	12.0	10.9	17.6
Unk3	−135.1*	−130.8*	7.8	21.3	11.4	13.6
Unk4	−55.4*	41.9*	92.0*	13.5	10.6	12.4
Unk5	171.8*	112.7*	−114.5*	14.9	50.1	159.5
Unk6	−106.7*	0.6	107.1*	29.7	45.7	35.5

The percentage difference was calculated based on the average values of relative signal integrals in each group. The calculations were made from left to right

*p < 0.01 using the Mann–Whitney–Wilcoxon test

**Fig. 5:**
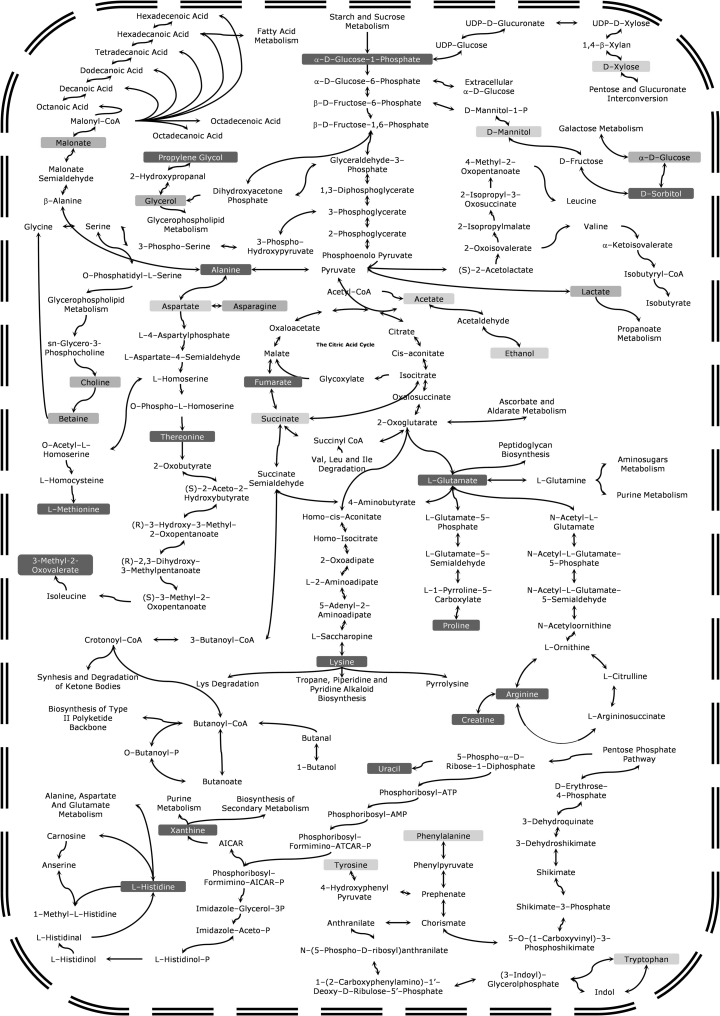
The general scheme of the metabolic pathways of *A. pallidofulvus* (*orange* boxes), *F. oxysporum* (*yellow* boxes) and *G. candidum* (*red* boxes). (Color figure online)

The most important metabolites correlated with the tested fungal genus were demonstrated in a biplot (Fig. 2b). Additionally, the correlation coefficient (r) was calculated for each comparison (Table 2). Taking into account the variables and their correlation coefficients, in positive correlation r > 0.9, and for each genus the characteristic metabolites were selected. The species of *A. pal* is represented by the highest concentrations of glycerol, glucitol and Unk5 (Fig. 5; bold italics metabolites in Table 2).

**Table 2:** Correlation coefficients (r) of intracellular primary endo-metabolites

Metabolite	*A. pal* vs. *F. oxy*	*A. pal* vs. *G. can*	*F. oxy* vs. *G. can*
Leucine	0.61	−0.88	−0.91
Valine	0.69	0.61	−0.37
Isoleucine	0.74	−0.50	−0.85
Isobutyrate	0.97	0.95	−0.86
**3-Methyl-2-oxovalerate**	0.92	0.96	0.87
*Propylene glycol*	0.99	0.29	−0.99
*Ethanol*	0.98	0.08	−0.98
Lactate	−0.05	−0.22	−0.11
Alanine	0.97	0.96	0.17
Arginine	0.74	0.84	0.41
Lysine	0.80	0.95	0.87
Acetate	0.93	0.94	−0.70
Proline	−0.41	0.88	0.89
Methionine	0.43	0.79	0.59
*4-Aminobutyrate*	0.97	−0.99	−0.99
**Glutamate**	0.48	0.96	0.96
**Pyruvate**	0.93	0.96	0.90
*Succinate*	0.97	0.73	−0.92
**Glutamine**	−0.43	0.97	0.97
**Citrate**	−0.37	0.97	0.97
2-Oxoisocaproate	0.39	0.89	0.87
Malate	0.94	0.88	0.42
Aspartate	0.81	−0.69	−0.89
Asparagine	−0.66	0.37	0.69
Creatine	0.11	0.85	0.94
Malonate	−0.98	−0.86	0.81
Choline	−0.63	0.15	0.54
sn-Glycero-3-phosphocholine	−0.91	−0.36	0.81
*myo*-Inositol	−0.94	0.00	0.51
***Glycerol***	−0.98	−0.98	−0.82
***Glucitol***	−0.98	−0.94	−0.33
Mannitol	0.34	−0.99	−0.99
Betaine	−0.77	−0.89	−0.44
Threonine	0.70	0.80	0.12
*Xylose*	0.98	−0.83	−0.99
Glucose	−0.96	−0.57	0.95
Glucose-1-phosphate	0.93	0.89	0.51
Uracil	0.83	0.94	0.54
Guanosine	0.90	−0.89	−0.97
Fumarate	0.78	0.96	0.90
Tyrosine	0.60	0.65	−0.02
Histidine	0.86	0.91	0.82
Phenylalanine	0.73	−0.29	−0.82
Tryptophan	0.81	0.71	−0.47
Xanthine	0.90	0.92	0.76
*Unk1*	0.98	0.17	−0.98
Unk2	0.03	−0.27	−0.24
Unk3	0.97	0.98	−0.31
*Unk4*	0.91	−0.86	−0.96
***Unk5***	−0.96	−0.93	0.63
Unk6	0.82	−0.01	−0.81

The positively correlated metabolites in *F. oxy* species were propylene glycol, ethanol, 4-aminobutyrate, succinate, xylose, Unk1 and Unk4 (Fig. 2b; italics metabolites in Table 2). In *G. can*, 3-methyl-2-oxovalerate, glutamate, pyruvate, glutamine and citrate exhibited positive correlation (Fig. 2b; bold metabolites in Table 2).

## Discussion

There is no evidence that previously published papers have comparatively analyzed the metabolite profiles of filamentous fungal pathogens including *A. pallidofulvus, F. oxysporum* and *G. candidum*. Our results show that a simple comparison of primary endo-metabolites using ^1^H NMR-based metabolomics can clearly separate these tested fungi. This is contradictory to most previous reports that postulated the lower power of primary endo-metabolites in chemotaxonomy and the differentiation of microorganisms (Frisvad et al. 2008; Andersen et al. 2008; Frisvad 1992; Jennessen et al. 2005). It is well-known that satisfactory growth of fungi and hence the production of metabolites is strongly dependent on the medium type and growth conditions (Frisvad et al. 2008; Andersen et al. 2001, 2003; Thrane 1993). Therefore, four types of media are routinely used in metabolic studies of fungi including the following: dichloran Rose Bengal yeast extract sucrose agar, malt extract agar, yeast extract sucrose (YES) agar, and potato dextrose agar (PBA) (Andersen et al. 2001, 2003; Thrane 1993). In this study, a medium based on potato-dextrose was used.

Biodiversity in fungal primary endo-metabolites showed that the three pathogenic strains, *A. pal, F. oxy* and *G. can*, are characterized by some particular variations in their fungal primary endo-metabolites despite the same growth conditions and general similarities in metabolism. The main differences are assigned in Fig. 5, where on a simple map of the biochemical pathways, increased concentrations of various metabolites can be seen for *A. pal* (orange boxes), *F. oxy* (yellow boxes) and *G. can* (red boxes).

### Biodiversity of the metabolic pathways

In comparison to *F. oxy* and *G. can*, the pathogenic strains of *A. pal* are characterized by some elevated metabolite levels included in galactose metabolism, namely glucitol (*d*-sorbitol) and *d*-glucose. Sorbitol is a sugar alcohol that arises from sucrose or glucose and fructose but rarely from glucose or fructose alone (Baek et al. 2010). The formation of sorbitol is often related to protecting cells against osmotic stress (Yoo and Lee 1993; Shen et al. 1999). Thus, sorbitol acts as high-osmotic pressure metabolite as well as protecting against protein denaturation. However, it is well-known that sorbitol is a component of potato-dextrose medium. Therefore, this finding might also be associated with the better uptake of sorbitol by *A. pal* cells from the growth medium than with its production in biochemical pathways.

The second group of metabolic pathways where strains of *A. pal* revealed higher concentrations of some metabolites is glycerophospholipid metabolism. Choline, sn-glycero-3-phosphocholine, glycerol and malonate are involved in the lipid biosynthesis necessary to form cellular membranes. Glycerol can be utilized for the backbone of different lipids as well as playing a crucial role in osmoregulation and maintaining a proper anabolic reduction charge (Shen et al. 1999; Vries et al. 2003; Clark et al. 2003). Sn-glycero-3-phosphocholine and choline as a source of methyl groups can take part in the elongation of glycerol- and glycerophospholipid chains. Additionally, betaine is a precursor for choline synthesis.

Malonate is a simple three-carbon dicarboxylic acid well-known to be a competitive inhibitor of succinate dehydrogenase (Kim 2002). The role of malonate is unclear, but its main objectives are related to nitrogen metabolism (Kim 2002). In plants, malonate has been suggested as a defensive metabolite when under stress.

Therefore, *A. pal* malonate might play a different protective role, and in addition, malonate might be included in the fatty acid biosynthetic pathways (components of cell wall and/or membrane).

Analysis of the *F. oxy* primary endo-metabolites resulted in the observation that the main differences are associated with a check-point of the respiration process. In oxidative respiration, pyruvate is integrated via acetyl-CoA into the citric acid cycle. When the oxygen level becomes insufficient, pyruvate is metabolized to ethanol via acetate. Thus, the elevated levels of ethanol and acetate in the cells of *F. oxy* suggest that this fungus can switch its metabolism to direct anaerobic respiration. However, the level of succinate (main intermediate of oxidative respiration) was also elevated. Therefore, it could be assumed that *F. oxysporum* metabolizes pyruvate both via the citric acid cycle and ethanol production.

In *F. oxy* higher concentrations of aspartate, leucine, isoleucine and isobutyrate, the primary metabolites for protein metabolism, anabolism (biosynthesis) and catabolism (degradation), could be noted. Additionally, phenylalanine is increased in *F. oxy* and can be synthesized by the interconversion of *d*-xylose (up-regulated in *F. oxy*) via the pentose phosphate pathway. Propylene glycol is known as a competitive inhibitor of glycerol transport (Castro and Loureiro-Dias 1991). Thus, it seems that *F. oxy* accumulated their metabolites more from direct protein synthesis than lipids and lipid components. However, the regulation of cell wall growth might be controlled by an elevated concentration of guanosine.

Among all of the discussed pathogenic filamentous fungi, *G. can* were characterized by highly increased levels of metabolites involved in oxidative respiration. Higher concentrations of metabolites of the citric acid cycle (citrate and fumarate) were noted. The excess of citrate can be utilized in the synthesis of glutamate and lysine (both in higher concentrations in the case of *G. can*) (Kanehisa et al. 2017). Glutamate can then be metabolized towards glutamine (up-regulated in *G. can*), which might be used to produce purine, aminosugars and proline (up-regulated in *G. can*). Glutamate can also be utilized in the urea cycle that plays a pivotal role in the N-metabolism of fungi, which was observed as the level of creatine was up-regulated (Kanehisa et al. 2017).

Generally, fungi are capable of producing different secondary metabolites such as antibiotics, volatile compounds and others, which is related to the activation of different metabolic pathways involving the biosynthetic precursors of these reactions (Zhai et al. 2017; Alberti et al. 2017). In *G. can*, the levels of histidine and xanthine, the main precursor metabolites in the biosynthesis of secondary metabolites, were elevated. Thus, it seems that *G. can* switch their metabolism towards the production secondary metabolites more so than *A. pal* and *F. oxy*.

In *G. can*, the levels of two α-keto acids, 3-methyl-2-oxovalerate and 2-osoisocaproate, were elevated. These metabolites, unique for fungal metabolism, are derived from amino acid degradation (Kanehisa et al. 2017). However, detailed analysis and their role in comparing the tested species is still unclear.

### Filamentous fungal virulence

The comparative primary endo-metabolome analysis of the three common filamentous fungi *A. pal, F. oxy* and *G. can* showed the main differences, but on this basis, it could also indicate the most dangerous fungal pathogens. These results suggest that *A. pal* is the most dangerous of the three, which is in agreement with previous reports. What makes *A. pal* a successful pathogen? In its metabolism can be observed the targeting metabolism on the protection of cell wall. Most antifungal agents act against the integrity of the cell wall. *A. pal*, by the synthesis of various components of lipids and the cell wall, might be able to resist these compounds. Additionally, the level of mannitol in *A. pal* is interesting. The concentration of mannitol is similar to that in *F. oxy*, and the HCA plot showed closer to grouping these two fungi. However, mannitol plays a very important role in the fungi as its presence allow cells to increase resistance and their virulence factors (Krahulec et al. 2011; Calmes et al. 2013; Ruijter et al. 2003).

## Conclusion

In this study the ^1^H NMR-based metabolomics approach was applied for the analysis of the biodiversity of filamentous fungal pathogens. According to our preliminary results, each of the tested strain (*A. pallidofulvus, F. oxysporum* and *G. candidum*) cultured in the same growth conditions revealed a specific metabolite profile. Moreover, we demonstrated that targeted metabolomics analysis could be utilized after careful optimization by other *omics* as well as biochemical assays in the future as a supporting taxonomical tool for currently methods.
